# Supplementary material for: Multidimensional Demographic Analyses of COVID-19 Vaccine Inequality in the United States: A Systematic Review
Source: Healthcare (Basel). 2025 Jan 13;13(2):139. doi: 10.3390/healthcare13020139 (PMC11765134; doi:10.3390/healthcare13020139)
Supplement: Supplementary file 1 [file healthcare-13-00139-s001.zip › healthcare-3392680-supplementary.pdf]

Methods Appendix

Multidimensional Demographic Analyses of COVID-19 Vaccine Inequality in the United States: A Systematic Review

Embase Search String:

('united states':ab,ti OR 'u.s.':ab,ti OR 'u.s.a':ab,ti OR 'us':ab,ti OR 'usa':ab,ti OR 'alabama':ab,ti OR 'al':ab,ti OR 'alaska':ab,ti OR 'ak':ab,ti OR 'arizona':ab,ti OR 'az':ab,ti OR 'arkansas':ab,ti OR 'ar':ab,ti OR 'california':ab,ti OR 'ca':ab,ti OR 'colorado':ab,ti OR 'co':ab,ti OR 'connecticut':ab,ti OR 'ct':ab,ti OR 'delaware':ab,ti OR 'de':ab,ti OR 'district of columbia':ab,ti OR 'florida':ab,ti OR 'fl':ab,ti OR 'georgia':ab,ti OR 'ga':ab,ti OR 'hawaii':ab,ti OR 'hi':ab,ti OR 'idaho':ab,ti OR 'id':ab,ti OR 'illinois':ab,ti OR 'il':ab,ti OR 'indiana':ab,ti OR 'iowa':ab,ti OR 'ia':ab,ti OR 'kansas':ab,ti OR 'ks':ab,ti OR 'kentucky':ab,ti OR 'ky':ab,ti OR 'louisiana':ab,ti OR 'la':ab,ti OR 'maine':ab,ti OR 'me':ab,ti OR 'maryland':ab,ti OR 'md':ab,ti OR 'massachusetts':ab,ti OR 'ma':ab,ti OR 'michigan':ab,ti OR 'mi':ab,ti OR 'minnesota':ab,ti OR 'mn':ab,ti OR 'mississippi':ab,ti OR 'ms':ab,ti OR 'missouri':ab,ti OR 'mo':ab,ti OR 'montana':ab,ti OR 'mt':ab,ti OR 'nebraska':ab,ti OR 'ne':ab,ti OR 'nevada':ab,ti OR 'nv':ab,ti OR 'new hampshire':ab,ti OR 'nh':ab,ti OR 'new jersey':ab,ti OR 'nj':ab,ti OR 'new mexico':ab,ti OR 'nm':ab,ti OR 'new york':ab,ti OR 'ny':ab,ti OR 'north carolina':ab,ti OR 'nc':ab,ti OR 'north dakota':ab,ti OR 'nd':ab,ti OR 'ohio':ab,ti OR 'oh':ab,ti OR 'oklahoma':ab,ti OR 'ok':ab,ti OR 'oregon':ab,ti OR 'or':ab,ti OR 'pennsylvania':ab,ti OR 'pa':ab,ti OR 'rhode island':ab,ti OR 'ri':ab,ti OR 'south carolina':ab,ti OR 'sc':ab,ti OR 'south dakota':ab,ti OR 'sd':ab,ti OR 'tennessee':ab,ti OR 'tn':ab,ti OR 'texas':ab,ti OR 'tx':ab,ti OR 'utah':ab,ti OR 'ut':ab,ti OR 'vermont':ab,ti OR 'vt':ab,ti OR 'virginia':ab,ti OR 'va':ab,ti OR 'washington dc':ab,ti OR 'dc':ab,ti OR 'washington':ab,ti OR 'wa':ab,ti OR 'west virginia':ab,ti OR 'wv':ab,ti OR 'wisconsin':ab,ti OR 'wi':ab,ti OR 'wyoming':ab,ti OR 'wy':ab,ti)

AND

('covid':ab,ti OR 'covid19':ab,ti OR 'corona':ab,ti OR 'covid-19':ab,ti OR '2019-ncov':ab,ti OR 'coronavirus':ab,ti OR 'sars-cov-2':ab,ti OR 'severe acute respiratory syndrome coronavirus 2':ab,ti)

AND

('vaccine':ab,ti OR 'vaccination':ab,ti OR 'vaccinated':ab,ti OR 'immunization':ab,ti OR 'immunisation':ab,ti OR 'injection':ab,ti OR 'injected':ab,ti)

AND

('administration':ab,ti OR 'receipt':ab,ti OR 'coverage':ab,ti OR 'rate':ab,ti OR 'rates':ab,ti OR 'service':ab,ti OR 'uptake':ab,ti OR 'completion':ab,ti OR 'first dose':ab,ti OR 'first-dose':ab,ti OR 'dose one':ab,ti OR 'dose-one':ab,ti OR 'second dose':ab,ti OR 'second-dose':ab,ti OR 'dose two':ab,ti OR 'partially vaccinated':ab,ti OR 'partially-vaccinated':ab,ti OR 'fully vaccinated':ab,ti OR 'fully-vaccinated':ab,ti OR 'booster':ab,ti OR 'boosted':ab,ti)

AND

('demographic':ab,ti OR 'sociodemographic':ab,ti OR 'socio-demographic':ab,ti OR 'socioeconomic':ab,ti OR 'socio-economic':ab,ti OR 'social class':ab,ti OR 'race':ab,ti OR 'racial':ab,ti OR 'ethnicity':ab,ti OR 'ethnic':ab,ti OR 'race/ethnicity':ab,ti OR 'racial/ethnic':ab,ti OR 'racial-ethnic':ab,ti OR 'black-white':ab,ti OR 'black/white':ab,ti OR 'black':ab,ti OR 'blacks':ab,ti OR 'african american':ab,ti OR 'african american/black':ab,ti OR 'hispanic':ab,ti OR 'latino':ab,ti OR

'latinos':ab,ti OR 'latinx':ab,ti OR 'hispanic/latino':ab,ti OR 'bipoc':ab,ti OR 'indigenous':ab,ti OR 'sex':ab,ti OR 'gender':ab,ti OR 'age':ab,ti OR 'aged':ab,ti OR 'geriatric':ab,ti OR 'age-group':ab,ti OR 'age group':ab,ti OR 'elderly':ab,ti OR 'medicare':ab,ti OR 'child':ab,ti OR 'children':ab,ti OR 'adolescent':ab,ti OR 'pediatric':ab,ti)

AND

('equity':ab,ti OR 'equality':ab,ti OR 'equitable':ab,ti OR 'inequity':ab,ti OR 'inequities':ab,ti OR 'inequality':ab,ti OR 'inequalities':ab,ti OR 'unequal':ab,ti OR 'disparity':ab,ti OR 'disparities':ab,ti OR 'healthcare disparities':ab,ti OR 'discrimination':ab,ti OR 'community vulnerability':ab,ti OR 'disadvantage':ab,ti OR 'disproportionate':ab,ti OR 'gap':ab,ti OR 'difference':ab,ti OR 'cluster':ab,ti OR 'clustering':ab,ti OR 'factors':ab,ti)

Pubmed Search String:

(("United States"[Title/Abstract] OR "U.S."[Title/Abstract] OR "US"[Title/Abstract] OR "U.S.A"[Title/Abstract] OR "USA"[Title/Abstract] OR "Alabama"[Title/Abstract] OR "AL"[Title/Abstract] OR "Alaska"[Title/Abstract] OR "AK"[Title/Abstract] OR "Arizona"[Title/Abstract] OR "AZ"[Title/Abstract] OR "Arkansas"[Title/Abstract] OR "AR"[Title/Abstract] OR "California"[Title/Abstract] OR "CA"[Title/Abstract] OR "Colorado"[Title/Abstract] OR "CO"[Title/Abstract] OR "Connecticut"[Title/Abstract] OR "CT"[Title/Abstract] OR "Delaware"[Title/Abstract] OR "DE"[Title/Abstract] OR "district of columbia"[Title/Abstract] OR "DC"[Title/Abstract] OR "Florida"[Title/Abstract] OR "FL"[Title/Abstract] OR "Georgia"[Title/Abstract] OR "GA"[Title/Abstract] OR "Hawaii"[Title/Abstract] OR "HI"[Title/Abstract] OR "imago"[Title/Abstract] OR "ID"[Title/Abstract] OR "Illinois"[Title/Abstract] OR "IL"[Title/Abstract] OR "Indiana"[Title/Abstract] OR "IN"[Title/Abstract] OR "Iowa"[Title/Abstract] OR "IA"[Title/Abstract] OR "Kansas"[Title/Abstract] OR "KS"[Title/Abstract] OR "Kentucky"[Title/Abstract] OR "KY"[Title/Abstract] OR "Louisiana"[Title/Abstract] OR "LA"[Title/Abstract] OR "Maine"[Title/Abstract] OR "ME"[Title/Abstract] OR "Maryland"[Title/Abstract] OR "MD"[Title/Abstract] OR "Massachusetts"[Title/Abstract] OR "MA"[Title/Abstract] OR "Michigan"[Title/Abstract] OR "MI"[Title/Abstract] OR "Minnesota"[Title/Abstract] OR "MN"[Title/Abstract] OR "Mississippi"[Title/Abstract] OR "MS"[Title/Abstract] OR "Missouri"[Title/Abstract] OR "MO"[Title/Abstract] OR "Montana"[Title/Abstract] OR "MT"[Title/Abstract] OR "Nebraska"[Title/Abstract] OR "NE"[Title/Abstract] OR "Nevada"[Title/Abstract] OR "NV"[Title/Abstract] OR "new hampshire"[Title/Abstract] OR "NH"[Title/Abstract] OR "new jersey"[Title/Abstract] OR "NJ"[Title/Abstract] OR "new mexico"[Title/Abstract] OR "NM"[Title/Abstract] OR "new york"[Title/Abstract] OR "NY"[Title/Abstract] OR "north carolina"[Title/Abstract] OR "NC"[Title/Abstract] OR "north dakota"[Title/Abstract] OR "ND"[Title/Abstract] OR "Ohio"[Title/Abstract] OR "OH"[Title/Abstract] OR "Oklahoma"[Title/Abstract] OR "OK"[Title/Abstract] OR "Oregon"[Title/Abstract] OR "OR"[Title/Abstract] OR "Pennsylvania"[Title/Abstract] OR "PA"[Title/Abstract] OR "rhode island"[Title/Abstract] OR "RI"[Title/Abstract] OR "south carolina"[Title/Abstract] OR "SC"[Title/Abstract] OR "south dakota"[Title/Abstract] OR "SD"[Title/Abstract] OR "Tennessee"[Title/Abstract] OR "TN"[Title/Abstract] OR "Texas"[Title/Abstract] OR "TX"[Title/Abstract] OR "Utah"[Title/Abstract] OR "UT"[Title/Abstract] OR "vermont"[Title/Abstract] OR "VT"[Title/Abstract] OR "Virginia"[Title/Abstract] OR "VA"[Title/Abstract] OR "washington dc"[Title/Abstract] OR "DC"[Title/Abstract] OR "Washington"[Title/Abstract] OR "WA"[Title/Abstract] OR "west virginia"[Title/Abstract] OR "WV"[Title/Abstract] OR "Wisconsin"[Title/Abstract] OR "WI"[Title/Abstract] OR "Wyoming"[Title/Abstract] OR "WY"[Title/Abstract])

AND

("covid"[Title/Abstract] OR "covid19"[Title/Abstract] OR "corona"[Title/Abstract] OR "covid-19"[Title/Abstract] OR "2019-nCoV"[Title/Abstract] OR "coronavirus"[Title/Abstract] OR "SARS-CoV-2"[Title/Abstract] OR "severe acute respiratory syndrome coronavirus 2"[Title/Abstract])

AND

("vaccine"[Title/Abstract] OR "vaccination"[Title/Abstract] OR "vaccinated"[Title/Abstract] OR "immunization"[Title/Abstract] OR "immunisation"[Title/Abstract] OR "injection"[Title/Abstract] OR "injected"[Title/Abstract])

AND

("administration"[Title/Abstract] OR "receipt"[Title/Abstract] OR "coverage"[Title/Abstract] OR "rate"[Title/Abstract] OR "rates"[Title/Abstract] OR "service"[Title/Abstract] OR "uptake"[Title/Abstract] OR "completion"[Title/Abstract] OR "first-dose"[Title/Abstract] OR "first dose"[Title/Abstract] OR "dose-one"[Title/Abstract] OR "dose one"[Title/Abstract] OR "second-dose"[Title/Abstract] OR "second dose"[Title/Abstract] OR "dose two"[Title/Abstract] OR "partially-vaccinated"[Title/Abstract] OR "partially vaccinated"[Title/Abstract] OR "fully-vaccinated"[Title/Abstract] OR "fully vaccinated"[Title/Abstract] OR "booster"[Title/Abstract] OR "boosted"[Title/Abstract])

AND

("demographic"[Title/Abstract] OR "sociodemographic"[Title/Abstract] OR "socio-demographic"[Title/Abstract] OR "socioeconomic"[Title/Abstract] OR "socio-economic"[Title/Abstract] OR "social class"[Title/Abstract] OR "race"[Title/Abstract] OR "racial"[Title/Abstract] OR "ethnicity"[Title/Abstract] OR "ethnic"[Title/Abstract] OR "race ethnicity"[Title/Abstract] OR "racial-ethnic"[Title/Abstract] OR "racial/ethnic"[Title/Abstract] OR "black-white"[Title/Abstract] OR "black/white"[Title/Abstract] OR "black"[Title/Abstract] OR "blacks"[Title/Abstract] OR "african american"[Title/Abstract] OR "african american black"[Title/Abstract] OR "Hispanic"[Title/Abstract] OR "Latino"[Title/Abstract] OR "Latinos"[Title/Abstract] OR "Latinx"[Title/Abstract] OR "hispanic latino"[Title/Abstract] OR "BIPOC"[Title/Abstract] OR "indigenous"[Title/Abstract] OR "sex"[Title/Abstract] OR "gender"[Title/Abstract] OR "age"[Title/Abstract] OR "aged"[Title/Abstract] OR "geriatric"[Title/Abstract] OR "age-group"[Title/Abstract] OR "age group"[Title/Abstract] OR "elderly"[Title/Abstract] OR "Medicare"[Title/Abstract] OR "child"[Title/Abstract] OR "children"[Title/Abstract] OR "adolescent"[Title/Abstract] OR "pediatric"[Title/Abstract])

AND

("equity"[Title/Abstract] OR "equality"[Title/Abstract] OR "equitable"[Title/Abstract] OR "inequity"[Title/Abstract] OR "inequities"[Title/Abstract] OR "inequality"[Title/Abstract] OR "inequalities"[Title/Abstract] OR "unequal"[Title/Abstract] OR "disparity"[Title/Abstract] OR "disparities"[Title/Abstract] OR "healthcare disparities"[Title/Abstract] OR "discrimination"[Title/Abstract] OR "community vulnerability"[Title/Abstract] OR "disadvantage"[Title/Abstract] OR "disproportionate"[Title/Abstract] OR "gap"[Title/Abstract] OR "difference"[Title/Abstract] OR "cluster"[Title/Abstract] OR "clustering"[Title/Abstract] OR "factors"[Title/Abstract]))

Web of Science Search String:

For Abstract Searches:
(((((AB=(United States OR U.S. OR US OR U.S.A OR USA OR Alabama OR AL OR Alaska OR AK OR Arizona OR AZ OR Arkansas OR AR OR California OR CA OR Colorado OR CO OR Connecticut OR CT OR Delaware OR DE OR District of Columbia OR DC OR Florida OR FL OR Georgia OR GA OR Hawaii OR HI OR Idaho OR ID OR Illinois OR IL OR Indiana OR IN OR Iowa OR IA OR Kansas OR KS OR Kentucky OR KY OR Louisiana OR LA OR Maine OR ME OR Maryland OR MD OR Massachusetts OR MA OR Michigan OR MI OR Minnesota OR MN OR Mississippi OR MS OR Missouri OR MO OR Montana OR MT OR Nebraska OR NE OR Nevada OR NV OR New Hampshire OR NH OR New Jersey OR NJ OR New Mexico OR NM OR New York OR NY OR North Carolina OR NC OR North Dakota OR ND OR Ohio OR OH OR Oklahoma OR OK OR Oregon OR OR OR Pennsylvania OR PA OR Rhode Island OR RI OR South Carolina OR SC OR South Dakota OR SD OR Tennessee OR TN OR Texas OR TX OR Utah OR UT OR vermont OR VT OR Virginia OR VA OR Washington OR WA OR West Virginia OR WV OR Wisconsin OR WI OR Wyoming OR WY))

AND

AB= (covid19 OR corona OR covid-19 OR SARS-CoV-2))

AND

AB= (vaccine OR vaccination OR vaccinated OR immunization OR immunisation OR injection OR injected)) AND AB = (administration OR receipt OR coverage OR rate OR service OR uptake OR completion OR first dose OR first-dose OR dose one OR dose-one OR second dose OR second-dose OR dose two OR full OR fully OR booster OR booster dose))

AND

AB = (race OR racial OR ethnicity OR ethnic OR race/ethnicity OR racial/ethnic OR racial-ethnic OR black-white OR black/white OR black OR African American OR African American/black OR Hispanic OR Latino OR Hispanic/Latino OR sex OR gender OR age OR age-group OR age group))

AND

AB =(equity OR equality OR equitable OR inequity OR inequities OR inequality OR inequalities OR disparity OR disparities OR discrimination OR gap OR difference OR cluster OR clustering)

For Title Searches:
(((((TI=(United States OR U.S. OR US OR U.S.A OR USA OR Alabama OR AL OR Alaska OR AK OR Arizona OR AZ OR Arkansas OR AR OR California OR CA OR Colorado OR CO OR Connecticut OR CT OR Delaware OR DE OR District of Columbia OR DC OR Florida OR FL OR Georgia OR GA OR Hawaii OR HI OR Idaho OR ID OR Illinois OR IL OR Indiana OR IN OR Iowa OR IA OR Kansas OR KS OR Kentucky OR KY OR Louisiana OR LA OR Maine OR

ME OR Maryland OR MD OR Massachusetts OR MA OR Michigan OR MI OR Minnesota OR MN OR Mississippi OR MS OR Missouri OR MO OR Montana OR MT OR Nebraska OR NE OR Nevada OR NV OR New Hampshire OR NH OR New Jersey OR NJ OR New Mexico OR NM OR New York OR NY OR North Carolina OR NC OR North Dakota OR ND OR Ohio OR OH OR Oklahoma OR OK OR Oregon OR OR OR Pennsylvania OR PA OR Rhode Island OR RI OR South Carolina OR SC OR South Dakota OR SD OR Tennessee OR TN OR Texas OR TX OR Utah OR UT OR vermont OR VT OR Virginia OR VA OR Washington OR WA OR West Virginia OR WV OR Wisconsin OR WI OR Wyoming OR WY))

AND

TI = (covid19 OR corona OR covid-19 OR SARS-CoV-2))

AND

TI = (vaccine OR vaccination OR vaccinated OR immunization OR immunisation OR injection OR injected))

AND

TI = (administration OR receipt OR coverage OR rate OR service OR uptake OR completion OR first dose OR first-dose OR dose one OR dose-one OR second dose OR second-dose OR dose two OR full OR fully OR booster OR booster dose))

AND

TI = (race OR racial OR ethnicity OR ethnic OR race/ethnicity OR racial/ethnic OR racial-ethnic OR black-white OR black/white OR black OR African American OR African American/black OR Hispanic OR Latino OR Hispanic/Latino OR sex OR gender OR age OR age-group OR age group))

AND

TI =(equity OR equality OR equitable OR inequity OR inequities OR inequality OR inequalities OR disparity OR disparities OR discrimination OR gap OR difference OR cluster OR clustering)

Proquest Search String:

ABSTRACT,TITLE(("United States" OR "U.S." OR "US" OR "U.S.A" OR "USA" OR "Alabama" OR "AL" OR "Alaska" OR "AK" OR "Arizona" OR "AZ" OR "Arkansas" OR "AR" OR "California" OR "CA" OR "Colorado" OR "CO" OR "Connecticut" OR "CT" OR "Delaware" OR "DE" OR "District of Columbia" OR "DC" OR "Florida" OR "FL" OR "Georgia" OR "GA" OR "Hawaii" OR "HI" OR "Idaho" OR "ID" OR "Illinois" OR "IL" OR "Indiana" OR "IN" OR "Iowa" OR "IA" OR "Kansas" OR "KS" OR "Kentucky" OR "KY" OR "Louisiana" OR "LA" OR "Maine" OR "ME" OR "Maryland" OR "MD" OR "Massachusetts" OR "MA" OR "Michigan" OR "MI" OR "Minnesota" OR "MN" OR "Mississippi" OR "MS" OR "Missouri" OR "MO" OR "Montana" OR "MT" OR "Nebraska" OR "NE" OR "Nevada" OR "NV" OR "New Hampshire" OR "NH" OR "New Jersey" OR "NJ" OR "New Mexico" OR "NM" OR "New York" OR "NY" OR "North Carolina" OR "NC" OR "North Dakota" OR "ND" OR "Ohio" OR "OH" OR "Oklahoma" OR "OK" OR "Oregon" OR "OR" OR "Pennsylvania" OR "PA" OR "Rhode Island" OR "RI" OR "South Carolina" OR "SC" OR "South Dakota" OR "SD" OR "Tennessee" OR "TN" OR "Texas" OR "TX" OR "Utah" OR "UT" OR "Vermont" OR "VT" OR "Virginia" OR "VA" OR "Washington" OR "WA" OR "West Virginia" OR "WV" OR "Wisconsin" OR "WI" OR "Wyoming" OR "WY")

AND

("covid19" OR "covid-19" OR "corona" OR "coronavirus" OR "SARS-CoV-2") AND ("vaccine" OR "vaccination" OR "vaccinated" OR "immunization" OR "immunisation" OR "injection" OR "injected")

AND

("administration" OR "receipt" OR "coverage" OR "rate" OR "service" OR "uptake" OR "completion" OR "first dose" OR "first-dose" OR "dose one" OR "dose-one" OR "second dose" OR "second-dose" OR "dose two" OR "full" OR "fully" OR "booster" OR "booster dose")

AND

("race" OR "racial" OR "ethnicity" OR "ethnic" OR "race/ethnicity" OR "racial/ethnic" OR "racial-ethnic" OR "black-white" OR "black/white" OR "black" OR "African American" OR "African American/black" OR "Hispanic" OR "Latino" OR "Hispanic/Latino" OR "sex" OR "gender" OR "age" OR "age-group" OR "age group" OR "minority" OR "minorities" OR "minority-group" OR "minority group" OR "vulnerability" OR "vulnerable groups" OR "underprivileged")

AND

("equity" OR "equality" OR "equitable" OR "equal" OR "inequity" OR "inequities" OR "inequality" OR "inequalities" OR "inequitable" OR "unequal" OR "disparity" OR "disparities" OR "discrimination" OR "gap" OR "difference" OR "cluster" OR "clustering" OR "disproportionate"))
